# Survival Rate, Biomechanical Complications, and Patient Satisfaction of Implant-Supported FRC Full-Arch Prostheses: A Retrospective Study with Follow up of 5 Years

**DOI:** 10.30476/dentjods.2023.98022.2047

**Published:** 2024-09-01

**Authors:** Daniel Hernández-González, Mauro Marincola, Antonio Díaz-Caballero, Alfredo Passaretti, Andrea Cicconetti

**Affiliations:** 1 Dept. of Prosthodontics, School of Dentistry, University of Cartagena, Columbia; 2 Implant Dental Center, School of Dentistry, University of Cartagena, Columbia; 3 Dept. of Periodontics, PhD in Biomedical Sciences, School of Dentistry, University of Cartagena, Columbia; 4 Dept. of Oral and Maxillofacial Sciences, School of Dentistry, Sapienza University of Rome, Italy

**Keywords:** Dental implants, Dental Prosthesis, Implant-supported, Survival Rate, Patient Outcome Assessment, Patient satisfaction

## Abstract

**Statement of the Problem:**

The satisfaction of patients with dentures on implants has different points of view that become fundamental aspects for the development of research on the quality of life of these patients, the eventual biomechanical complications to which these prostheses and implants can be subjected, and design considerations for cantilever extensions.

**Purpose:**

The objective of research was to assess the implants and prosthesis survival rates, biomechanical complications relative to the length of the distal extensions (cantilevers), and the satisfaction of the patients with a fixed implant-supported full-arch fiber-reinforced composites prosthesis

**Materials and Method:**

A retrospective clinical and radiographic cohort study was developed. Clinical records of a selected cohort were analyzed according to inclusion and exclusion criteria. Data on a patient who underwent to fixed implant-supported full-arch fiber-reinforced composites prosthesis at least of five years of function were collected. Data analysis was performed using Kaplan-Meier curves and Fisher's Exact Test. P values less than 0.05 were considered statistically significant

**Results:**

After insertion, 1 of 29 prostheses failed, the overall prosthetic survival rate observed at 5 years was 96.5%. Of the 120 implants placed in 28 patients, only 4 patients experienced loss of an implant during the 5 years of observation; the implant survival rate throughout the observation period was 86.2%. Distal extension seems to negatively affect the prognosis of implant-supported rehabilitation. Regarding the level of satisfaction of the patient with the prosthesis, none reported being uncomfortable or dissatisfied neither with their appearance nor with the taste of food throughout the studied period

**Conclusion:**

No relevant associations were found between the variables involved. The study found the improvement in quality of life following the installation of fixed rehabilitation on the patients. Once the potential benefits of patients are obtained, controlled clinical trials are encouraged.

## Introduction

Implant-supported full-arch fixed prostheses are well-studied long-term treatment for the edentulous patient [ 1 ]. A survival rate of 93.3% after 10 years and 87.1% after 20 years was presented by Chrcanovic *et al*. [ 1 ]. Chochlidakis *et al*. [ 2 ] reported possible biological and mechanical complications of the prosthesis. To avoid these potential complications and to achieve predictable long-term success with the full-arch implant-supported prosthesis, certain biomechanical concepts are important: the number and position of the implants [ 3 ], the extension of the cantilever (distal extension) [ 4 ], and the fabrication material of the prosthesis [ 5 ]. 

The number and position of the implants in an edentulous jaw varied in recent publications. The initial protocol proposed by Branemark [ 6 ] was six implants for the maxilla and five for the mandible. Recent systematic reviews and meta-analysis studies suggest four implants for the maxilla and three for the mandible show no statistical difference relative to five or more implants for each jaw [ 3 ]. 

The position and distribution of the implants was associated with distal cantilevers [ 4 ], which can biomechanically overload the prostheses causing biological or mechanical complications such as screw loosening, debonding, prosthesis delamination or fracture, peri-implantitis, and abutment or implant fracture. There is no a proven scientific formula for the length of cantilever to optimize the survival rate of the prosthesis; however, Purcell *et al*. [ 9 ] have postulated rules. For example, one considers the ratio between the anterior-posterior spread (AP spread) of the implants and the cantilever’s length (CL) to be significant. This ratio (AP/CL ratio) should be less than 2 [ 7 ]. 

Moreover, the material used for prosthesis fabrication is crucial for the biomechanical behavior of the implant-prosthetic restoration [ 5 ]. Originally, Branemark [ 6 ] proposed the use of cast metal reconstructions with acrylic resin teeth; however, with the CAD/CAM man-ufacturing technology, a wide range of materials such as Cr-Co alloys, titanium, zirconia and ceramics have been used [five]. McGlumphy *et al*. [ 8 ] demonstrated that there were frequent complications of acrylic fracturing or porcelain chipping, even with the use of minimal cantilever lengths (AP/CL ratio<1). Currently, there is rese-arch interest in fiber-reinforced composites (FCR), that have more flexible strength and significantly better bio-mechanical attributes. FRC is an elastic and anisotropic material, that can be deformed without being permanently distorted and can adsorb the occlusal loads even in case of long cantilever (AP/CL ratio>2) more than rigid materials like titanium or zirconia (AP/CL ratio < 1) [ 9 ].

Da Cunha *et al*. [ 9 ] have shown improved patient satisfaction, positive psychological behavior, and a better quality of life, when fixed implant-supported prostheses were used. ADDIN ZOTERO_ITEM CSL_CITATION {"citationID":"3fAHYnc4","properties":{"formattedCitation":"[ 12 ]","plainCitation":"[ 12 ]","dontUpdate":true,"noteIndex":0},"citationItems":,"itemData":{"id":891,"type":"article-journal","abstract":"AIM: To compare implant fixed complete dentures with implant overdentures relative to prosthodontic outcomes.\nMATERIAL AND METHODS: An electronic Medline (PubMed) with MeSH terms, and Cochrane library search was performed, focusing on studies that included implant fixed complete dentures and implant overdentures in the same study, with the results based on studies that included both types of prostheses.\nRESULTS: The following six categories of comparative studies were identified in the literature: 1) Implant and prosthesis survival; 2) Prosthesis maintenance/complications; 3) Bone changes; 4) Patient satisfaction and quality of life; 5) Cost-effectiveness; and 6) Masticatory performance. It was determined that both the fixed and removable treatments were associated with high implant survival rates. However, both types of prostheses were impacted by the need for post-placement mechanical maintenance or prosthetic complications. More maintenance/complications occurred with implant overdentures than with fixed complete dentures. Residual ridge resorption was greater with implant overdentures. Patient satisfaction was high with each prosthesis, with three studies revealing higher satisfaction with fixed complete dentures and five studies finding no difference. All but one study on cost-effectiveness indicated implant overdentures were more cost-effective. Based on two studies, it appears the masticatory performance of implant fixed complete dentures and implant overdentures is comparable.\nCONCLUSIONS: Multiple factors must be considered when determining whether an implant-fixed complete denture or implant overdentures are best suited for patients with completely edentulous jaws. Conflict-of-interest statement: The authors declare they have no conflicts of interest.","container-title":"European Journal of Oral Implantology","ISSN":"1756-2406","journalAbbreviation":"Eur J Oral Implantol","language":"eng","note":"PMID: 28944366","page":"13-34","source":"PubMed","title":"Fixed vs removable complete arch implant prostheses: A literature review of prosthodontic outcomes","title-short":"Fixed vs removable complete arch implant prostheses","volume":"10 Suppl 1","author":,"issued":{"date-parts":]}}},{"id":1177,"uris":,"itemData":{"id":1177,"type":"article-journal","abstract":"This study evaluated patients' expectation before and satisfaction after full-arch fixed implant-prosthesis rehabilitation. Other variables that could influence patient satisfaction with this therapy were also evaluated. Using a visual analog scale (VAS), a sample of 28 patients assigned scores for their expectation before and satisfaction after therapy regarding chewing, esthetics, comfort, and phonetics. They also completed a questionnaire concerning their evaluation of the dentists' conduct. The average VAS scores were high for both expectation prior to treatment and satisfaction after treatment, and there was no statistical difference between them. Women presented higher expectations than men regarding esthetics (P = 0.040), phonetics (P = 0.043) and comfort (P = 0.013). Significant differences were not found between VAS scores with clinical variables (arch, radiographic bone quality, surgical bone quality, and implant inclination), educational level, and patients' evaluation of the dentists' conduct. Considering the results obtained in this study, expectation before implant-supported, full-arch fixed prosthesis therapy were met following treatment, with women having higher expectations than men.","container-title":"Journal of Oral Implantology","DOI":"10.1563/AAID-JOI-D-12-00134","ISSN":"1548-1336, 0160-6972","issue":"3","language":"en","page":"235-239","source":"DOI.org (Crossref)","title":"Patients' Expectation Before and Satisfaction After Full-Arch Fixed Implant-Prosthesis Rehabilitation","volume":"41","author":,"issued":{"date-parts":]}}}],"schema":"https://github.com/citation-style-language/schema/raw/master/csl-citation.json"} The most common way to assess the perception of implant treatment is the Oral Health‐Related Quality of Life (OHRQoL) [ 12 ], although the Oral Health Impact Profile (OHIP) is the most widely accepted protocol [ 13 ]. The shorter version of this OHIP instrument with 5 questions is sufficient and may still be practical and informative [ 14 ]. The questionnaire assesses five macro-area, chewing, pain, appearance, food flavor, and personal limitation. This version has been translated in many languages; it is coming to be routinely used in clinical practice [ 15 ].

Accordingly, this study aimed to evaluate the survival rate of cantilever extension prostheses, biomechanical complications, and the satisfaction of patients with a fixed implant-supported full-arch fiber-reinforced composites prosthesis.

## Materials and Method

A retrospective clinical and radiographic cohort study was developed. An electronic and manual review of available clinical records was collected from patients treated between February of 2013 and November of 2016 at Dental School of Cartagena (Colombia) and Dental School of La Sapienza University of Rome (Italy). After this period, the evaluators started the following up for each patient.

The inclusion criteria for the participants were total edentulous patients restored with a FRC, full arch implant-supported fixed prostheses under functional loading at least 5 years, with ages between 18 and 80 years old. All patients were informed in detail about the objectives of the study and were given a written informed consent form according to the *Declaration of Helsinki*.

The data collected included gender (male/female), age (expressed in completed years), medical status (“healthy” and “mild” according to ASA classification); smoking habits (dichotomic, yes or not); opposing dentition (denture, natural teeth, fixed prosthesis, partial edentulous), distal extension (short: < than 21 mm and long: > than 21 mm). 

The clinical records were analyzed to assess (1) Prosthesis survival rate (prosthesis remaining in situ without modifications during the entire observation period), (2) Patient implant loss experience (the report of at least an event of implant loss in each patient), and (3) Biomechanical complications (screw loosening, debonding, prosthesis delamination or fracture, peri-implantitis, and abutment or implant fracture) according to Moraschini *et al*.’s study [ 16 ]. 

Technical processes were evaluated by at least two different team members. To avoid disparity in criteria, the decision of a third evaluator was relied upon to resolve any lack of uniformity.

The patient satisfaction was evaluated according to the OHIP5 questionnaire adapted to Spanish language by Simancas *et al*. [ 13 ] administered annually after prosthesis insertion in both Dental School. Responses were presented on a 5-point ordinal scale (0, never; 1, hardly ever; 2, occasionally; 3, fairly often; and 4, very often). Summing all responses resulted in a score ranging from a minimum of 0 to a maximum of 20. A larger score indicated a more negative impact of oral health problems. The technical processes were evaluated by at least two different team members. To avoid disparity in criteria, the decision of a third evaluator was relied upon to resolve any lack of uniformity.

Data analysis was performed using R version 4.02 for Windows. Descriptive statistics were calculated for categorical and numerical variables. Frequencies and percentages were obtained for categorical variables; numerical summaries were calculated to obtain means and standard deviations. Kaplan-Meier curves were generated to estimate the overall survival probability for prosthesis, patient experience of implant loss, and incidence of biomechanical complications. The estimation of the relationship between prosthesis survival, patient experience of implants loss, biomechanical complication cumulative incidence, and covariates were analyzed using the Fisher's Exact Test. P values less than 0.05 were considered statistically significant.

## Results

28 patients (13 males and 15 females) with 29 full arches fixed prostheses have met the selection criteria between the two dental schools. The average age was 67.8 years-old, 89.7% of patients were healthy, and 86.2% were non-smokers. All patients received at least one implant supported full-arch, cement-retained, metal-free rehabilitation made of FRC material (Trinia, Bicon, LL-C) veneered with a zirconium silicate micro ceramic material (Ceramage, Shofu, Fukuine, Japan). 18 patients had an opposing denture, 2 patients had partial edentulous dentition, 6 had completed natural teeth, and 3 had porcelain fused to metal fixed prosthesis. 24 prostheses were at the lower and 5 were at the upper. 21 patients belonged to the “short” distal extension group, while 8 to the “long” group. 27 prostheses were supported by 4 implants, while 2 were supported by 6 implants. The total number of implants placed in the population was 120. 

### Prosthesis survivor

**Figure 1:**
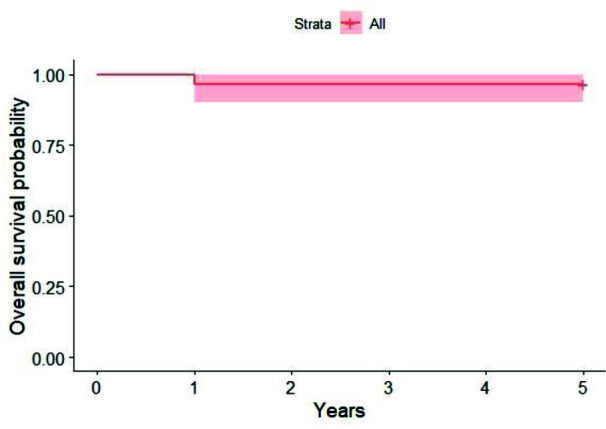
Kaplan- Meier curve estimates for prosthesis survival

After insertion, 1 of 29 prostheses failed in the “long” distal extension group, the overall prosthetic survival rate observed at the follow up was 96.5%. The only prosthetic failure was during the first year of the observation period (Figure 1).

### Patient implant loss experience

**Figure 2:**
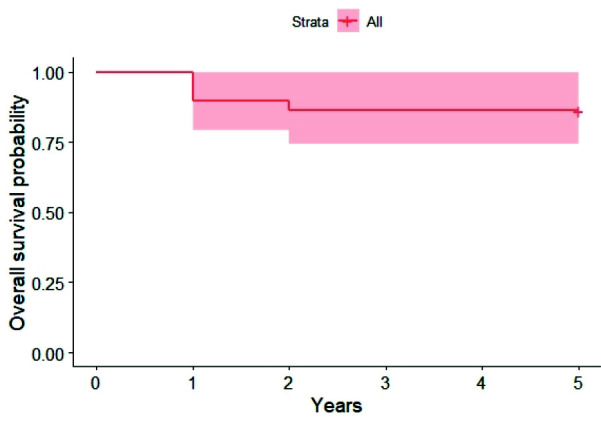
Kaplan- Meier curve estimates for patient implant loss experience survival

Of the 28 patients, only 4 patients (14.2%) experienced loss of at least one implant during the follow up period, while patients who did not experience any implant loss were 85.7%. The incidence of event “implant loss” during the 5 years of follow-up and the overall survival rate can be seen in Figure 2.

On 120 implants placed, 6 of them were lost in 4 patients during the follow up. Three patients belonged to the “long” distal extension group and one to the “short” distal extension group. The overall implant survival rate was 95%. All implant failure occurred in the first 2 years of follow up. 3 of the failed implants were lost in the same patient during the first year of follow-up. In the same period, two patients lost one implant each. While the last implant failed in a patient at two years of follow up.

### Biomechanical complications

There were recorded only 11 prosthetic complications among the population during the observation period. During the first year, there were 3 teeth dislodgements.

**Figure 3:**
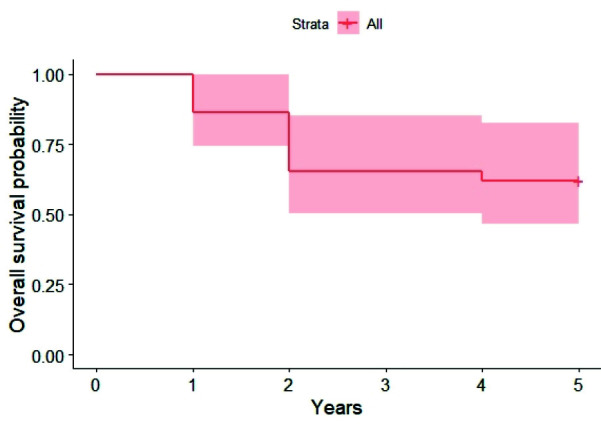
Kaplan- Meier curve estimates for biomechanical complications

Subsequently, there was 1 abutment fracture and decementation of the prosthesis from their implant abutments. The incidence of these biomechanical complications during the observation period is presented in Figure 3.

Of the 11-patient affected by biomechanical complications reported, 8 belonged to the “short” distal extension group, while 3 belonged to the “long” distal extension group. The biomechanical complications, prosthesis and implant survival have been placed in relation to the distal extension (See Table 1).

**Table 1:** Analysis of prosthesis survival, implant survival and biomechanical complications cumulative incidence according to study covariates

Variable	Prosthesis		*p* value	Implant		*p* value	Biomechanical complications		*p* value
	Survival n (%)	Failure n (%)		Survival n (%)	Failure n (%)		No n (%)	Yes n (%)	
Gender									
Female	16 (55.2)	0 (0)	0.4483	13 (44.8)	3 (10.3)	0.6059	10 (34.5)	6 (20.7)	1.000
Male	12 (41.4)	1 (3.4)		12 (41.4)	1 (3.4)		8 (27.6)	5 (17.2)	
General health									
Healthy	26 (89.7)	0 (0)	0.1034	24 (82.8)	2 (6.9)	0.0422	17 (58.6)	9 (31)	0.5394
Mild	2 (6.9)	1 (3.4)		1 (3.4)	2 (6.9)		1 (3.4)	2 (6.9)	
Medications									
No	22 (75.9)	0 (0)	0.2414	21 (72.4)	1 (3.4)	0.0339	15 (51.7)	7 (24.1)	0.3746
Yes	6 (20.7)	1 (3.4)		4 (13.8)	3 (10.3)		3 (10.3)	4 (13.8)	
Smoking									
No	24 (82.8)	1 (3.4)	1.000	21 (72.4)	4 (13.8)	1.000	16 (55.1)	9 (31)	0.6221
Yes	4 (13.8)	0 (0)		4 (13.8)	0 (0)		2 (6.9)	2 (6.9)	
Number of implants									
Four	26 (89.7)	1 (3.4)	1.000	24 (82.8)	3 (10.3)	0.2611	17 (58.6)	10 (34.5)	1.000
Six	2 (6.9)	0 (0)		1 (3.44)	1 (3.44)		1 (3.4)	1 (3.4)	
Distal extension									
Short	21 (72.4)	0 (0)	0.2759	20 (69)	1 (3.4)	0.0525	13 (44.8)	8 (27.6)	1.000
Long	7 (24.1)	1 (3.4)		5 (17.2)	3 (10.3)		5 (17.2)	3 (10.3)	

### Patients’ satisfaction

The OHIP questionnaire, administered annually in the two dental schools, allowed checking over the time the patient’s perception and satisfaction about the rehabilitation. Data are listed in Table 2; it is important to note that none of the patients reported to be uncomfortable or dissatisfied neither with their appearance nor with the taste of food throughout the studied period. Additionally, only in the first year, a difficulty during function with a mean punctuation of 0.34±1.04 was reported.

**Table 2:** Patient satisfaction

OHIP item	Follow-up				
	1 Mean (SD)	2 Mean (SD)	3 Mean (SD)	4 Mean (SD)	5 Mean (SD)
Difficulty in chewing	0.72 (1.22)	0.39 (0.87)	0.14 (0.59)	0.07 (0.38)	0.15 (0.53)
Painful aching	0.59 (1.2)	0.10 (0.31)	0.07 (0.37)	0.07 (0.38)	0.11 (0.42)
Uncomfortable with appearance	0 (0)	0 (0)	0 (0)	0 (0)	0 (0)
Less flavor in food	0 (0)	0 (0)	0 (0)	0 (0)	0 (0)
Difficulty doing usual jobs	0.34 (1.04)	0 (0)	0 (0)	0 (0)	0 (0)
Total score a	1.65 (3.15)	0.50 (1.03)	0.21 (0.95)	0.14 (0.76)	0.26 (0.94)

a Maximum total score = 20

The items “difficulty in chewing” and “painful aching” had a higher mean punctuation (0.72±1.22 and 0.59 ±1.2, respectively) at the first year, which decreased during the 5 years of follow-up (Table 2).

## Discussion

The main aim of this study was to assess the possible influence of the cantilever on performances of prosthetic rehabilitations made of fixed, full-arch, and implant supported FRC prosthesis in an average observational period of 5 years. Attention was given to patient perception and satisfaction.

The cumulative prosthetic survival rate was 96.5%. The overall implant surviving rate was 95%. 11 patients experienced at least one biomechanical complication.

In terms of expected results, the authors’ hypothesis was to find comparable outcomes of implant/prosthesis survival rate to previous reported studies by Meriç *et al*. [ 17 ], and Cicconetti *et al*. [ 18 ]. As it stands, the performance of implant-supported FRC-prostheses studied are comparable to the state of art for this kind of total-arch fixed rehabilitation [ 19 ].

With respect to the position of the implants and consequently, the length of the distal extensions (cantilevers), even with a numerical disparity between the two groups (21 short and 8 long), the only failure of prosthetic rehabilitation was observed in the long distal-extension group (see table 1). 4 patients experienced the loss of at least one implant during the observation period. 3 belonged to the “long” distal-extension group and 1 to the “short”. 11 patients experienced at least one biomechanical complication. Eight of these patients were enrrolled into the “short” distal-extension group and three of them into the “long” distal-extension group. The statistical analysis did not reveal any association between the three variables and the cantilever.

Analyzing data, 4 patients lost at least one implant but only a patient loses the prosthesis thus there are 3 patients with prosthesis working on a limited number of implants for at least 3 years. The biomechanical behavior of FRC prostheses could play a crucial role in the survival of these prostheses, because of its capability to bear loads and extensions.

Finally, in the present study, the perception of the patients was considered. It should be noted that, compared to Duong *et al*. [ 20 ], the quality of life increases during the years. It has already been stated by other authors that an improvement in masticatory ability and patient comfort results in an increase in self-esteem and personal security according to Fueki *et al*. [ 21 ], Haraldson *et al*. [ 22 ], and Strassburger *et al*. [ 23 ]. The data recorded on patient satisfaction indicate that the assessed population positively perceives the benefit of rehabilitation. On the population observed, we found a better performance of the treatment when administered to people with good general health and no intake of medication (p< 0.05). 

## Conclusion

According to the findings and within the limitations of the present retrospective clinical study, the cumulative survival rate of prostheses and implants was comparable to the previously reported study. Although no relevant associations were found between the variables involved, statistically relevant indications cannot be drawn except only the recommendation not to exceed 21 mm of cantilever. The study considered the improvement in quality of life following fixed rehabilitation. Once the potential benefits of patients are obtained, controlled clinical trials are encouraged. The properties of FRC materials can allow rehabilitations to an increasing number of patients, thanks also to the possible cantilever.
